# The Impact of Social Context and Language Comprehension on Behaviour: A Kinematic Investigation

**DOI:** 10.1371/journal.pone.0085151

**Published:** 2013-12-20

**Authors:** Claudia Gianelli, Luisa Lugli, Giulia Baroni, Roberto Nicoletti, Anna M. Borghi

**Affiliations:** 1 Division of Cognitive Sciences, University of Potsdam, Potsdam, Germany; 2 Department of Philosophy and Communication, University of Bologna, Bologna, Italy; 3 Department of Psychology, University of Bologna, Bologna, Italy; 4 Institute of Cognitive Sciences and Technologies, National Research Council, Roma, Italy; University of Leicester, United Kingdom

## Abstract

We investigated whether and how comprehending sentences that describe a social context influences our motor behaviour. Our stimuli were sentences that referred to objects having different connotations (e.g., attractive/ugly vs. smooth/prickly) and that could be directed towards the self or towards “another person” target (e.g., “The object is ugly/smooth. Bring it to you/Give it to another person”). Participants judged whether each sentence was sensible or non-sensible by moving the mouse towards or away from their body. Mouse movements were analysed according to behavioral and kinematics parameters. In order to enhance the social meaning of the linguistic stimuli, participants performed the task either individually (Individual condition) or in a social setting, in co-presence with the experimenter. The experimenter could either act as a mere observer (Social condition) or as a confederate, interacting with participants in an off-line modality at the end of task execution (Joint condition). Results indicated that the different roles taken by the experimenter affected motor behaviour and are discussed within an embodied approach to language processing and joint actions.

## Introduction

The ability to coordinate our actions with others is crucial for our species. Given our social nature, it is striking that cognitive scientists have focused so far more on individual cognition, rather than on collaborative activities. In the very last years, though, cognitive scientists have started to devote more attention to the social aspects of cognition [1–4]. A great impulse to this kind of research has been given by the discovery of the mirror neuron system [5], and by the development of common coding theories [6,7], which are both supported by a variety of data showing that humans rely on their own motor system while observing and predicting actions performed by others. The ability to collaborate with others, to take turns, to act in a coordinate and joint manner is necessary for language and communication as well. Recent studies have started to investigate joint action and language, considering dialogue as an interesting example of an integrated form of joint action [8,9]. It has to be pointed out, though, that even if these studies on verbal exchange have paved the way for current joint action research, they did not tackle the issue of “how lower-level processes like action simulation and higher-level processes like verbal communication and mental state attribution work in concert, and under which circumstances they can overrule each other” ([1], p. 365).

Studies on how the social context can impact language comprehension are of interest for embodied and grounded theories of cognition, according to which language comprehension implies the recruitment of the same perception, action, and emotion systems that are activated while interacting with the objects and while performing the actions language refers to [10–14]. In recent years, a large number of behavioural, neurophysiological and brain imaging studies have provided compelling evidence in favour of this view (for reviews, see 10,15–17). However, the majority of these studies have focused on simple action verbs, for instance kicking and grasping, and on nouns referring to concrete, manipulable objects, for instance cups and pans (for a review, see 18). Furthermore, the emotional and social context in which actions take place has been rarely considered [4,19]. 

A recent study by Lugli and co-authors [20] investigated the extent to which the social context may be conveyed by linguistic meaning in tasks involving written sentence comprehension. Participants were faced with sentences describing positive/negative and easy/difficult to grasp objects that could be directed towards the agent or towards other persons (i.e., “The object is nice/ugly/smooth/prickly. Bring it to you/Give it to another person/friend”). Participants’ task was to discriminate between sensible and non-sensible sentences (i.e., fillers) by moving the mouse towards or away from their body. The novelty of this paradigm was that the linguistically described objects were framed in a social perspective represented by the “Bring it to you/ Give it to another person/friend” actions and targets. The authors found that the influence of the social context failed to emerge when the target described in the sentence was not familiar enough (i.e., “another person”) to lead participants to properly simulate the social context (Experiment 1). Conversely, the social context influenced the motor behaviour when the target shared a familiar and positive relationship with the agent (e.g., “friend”, Experiment 2). Taken together, these results indicated that the written sentences evoked a motor simulation, which is modulated by the way the social context is linguistically described.

Two recent embodied theories of language try to cast light on the link between the simulation occurring during language comprehension and the context experienced by participants. The first account is the Indexical Theory [21], which proposes that words are indexed to their referents in the world. Hence, words referring to objects would evoke perceptual and also motor information related to those objects and would re-enact, through an instantiation mechanism, the perceptual and interactive experience we have with them. For example, in order to understand the sentence "He sweeps the floor with a toothbrush" we would index the words referents, which are represented in terms of perceptual symbols [22] and not in propositional terms. The affordances of words referents would then be derived and meshed in order to comprehend the sentence - in this case the sentence is strange but meaningful, since the affordances of a toothbrush are compatible with sweeping. According to this theory, words meaning is constrained by the affordances of words referents rather than by the associations between words and by word frequency, as distributional approaches assume [23]. 

The second account is the Action Based Language model (from now on ABL model, [24]), inspired by Wolpert's theory on motor control [25]. The ABL model proposes that, when we comprehend language, a prediction of the effects of the sensorimotor and emotional states is advanced. Wolpert's theory of motor control includes controllers (or backward models), which compute motor commands to accomplish goals, and predictors (or forward models) responsible for generating predictions of the effects of actions. According to the ABL model, in language comprehension both controllers and predictors would be activated. For example, upon hearing the verb “walk”, the mirror neuron system would activate an associated action controller responsible for generating motor commands. Later, the predictor of the word would generate possible outcomes of the action to perform. While both theories make use of the notion of simulation, the ABL model stresses the predictive role of it and gives more relevance to the importance of action for language comprehension with respect to the Indexical Theory. 

Understanding how the matching between the situations simulated during language comprehension and our experience occurs would be crucial for both theories. It is worth noting, though, that according to embodied and grounded theories the re-enactment evoked by linguistic stimuli represents a form of simulated experience. The degree at which this simulated experience shares aspects with our experience of objects and motor information varies in detail and depth. In this sense, Barsalou ([22], p. 1281) argues that: “re-enactments are always partial and potentially inaccurate”, and Jeannerod [12] clarifies that: “Simulating is not doing”. As a consequence, retrieving an action through linguistic stimuli would activate just partially the neural pattern evoked by the actual motor experience. 

The present study addressed how the presence of an observer or a confederate in the experimental setting can modify the simulation formed while comprehending sentences that describe an action occurring in a social context. Goal of this work was indeed to enhance the simulation of the social context linguistically described in the sentences by matching it with the actual social context. To this aim, we introduced two novelties with respect to Lugli et al.’s [20] study. First, we introduced an actual social setting: participants could perform the experiment alone (Individual condition), in presence of the experimenter (Social condition) or in presence of the experimenter acting as a confederate (Joint condition). More precisely, in the Social condition the experimenter sat in front of the participant throughout the whole task, while in the Joint condition the experimenter interacted with the participant at the end of the task execution. The stimuli and procedure used in these three conditions were identical to those used in Lugli et al.’s Experiment 1. Participants were faced with sentences describing the self and “another person” targets (e.g., “The objects is nice. Bring it to you/Give it to another person”) and were required to move the mouse towards/away from their body according to sentence sensibility (i.e., fillers vs. non-fillers). The similarity between the linguistically described target (“another person”) and the actual target (the experimenter, to whom participants have never spoke to or interacted with before) was expected to lead participants to simulate better and in a more accurate way the social context described in the sentence. 

The second novelty of the study consisted in the fact that kinematics measures were recorded together with reaction times (RTs). Kinematics analyses offer a detailed and ecological measure of sentence processing in a social context. Specifically, these measures allowed us to test how motor processes were influenced by the action-related language processing and by the social aspects of interaction. In particular, we expect kinematics measures to give fine-grained information on how different object properties and the social context may affect the execution of simple motor acts. Therefore, we focused in particular on the amplitude of velocity peaks, a well-known measure useful to detect linguistic effects at the stage of motor planning.

Our predictions were as follows:

1) Observer vs. confederate

We hypothesized that the presence of an actual target, that is the experimenter, could enhance the link between the linguistic stimuli and the motor system. In other words, the presence of the experimenter acting as an observer or as a confederate could allow participants to form a more detailed simulation of the linguistically described “another person” target. Participants, in fact, would be able to match the content of their simulation with an actual target (i.e., the experimenter). Specifically, and in line with the Indexical Theory, we predicted a more detailed simulation in the Social and Joint conditions compared to the Individual one, since the first two conditions could allow a direct indexing of the linguistically described target, while the third one could not. Furthermore, in line with the ABL model, which emphasizes the importance of action and of the predictive role of simulation for acting, we explicitly predicted an advantage of the Joint condition over the Social one. Our hypothesis was indeed that the simulation of the linguistically described “another person” target could be more detailed for the Joint condition, with respect to the Social and Individual ones, thus affecting both RTs and velocity peaks similarly to what happens when an actual social interaction takes place. This result would be in line with previous studies (e.g., 26,27) showing that when a precise motor act has to be performed with another person, a higher accuracy is required. Here, along with the kinematics literature, we intend accuracy as referred to movement execution, not to correctness of response. This higher accuracy and carefulness in movement execution can be detected through key kinematics parameters [26,27] such as the amplitude of velocity peaks. On this basis, we predicted a stronger modulation of the amplitude of velocity peaks in the Joint with respect to the Social condition. In particular, we expected lower velocity peaks and slower RTs in the Joint as compared to the Social and the Individual conditions.

2) Object properties: qualitative vs. grasp-related

In line with previous kinematics studies, we expected that grasp-related properties would be processed more accurately in the Joint compared to the Social condition, thus yielding lower velocity peaks and slower RTs, indicating higher accuracy requirements. Indeed, we expected increased accuracy requirements because in the Joint condition the presence of the experimenter had to be taken into account while performing both the linguistic (sentence comprehension and evaluation) and the motor task (moving the mouse towards or away from the body). 

## Methods

### Ethics Statement

All participants gave their written informed consent and the Ethics Committee of the Department of Psychology at the University of Bologna approved the study.

### Participants

Twenty-four undergraduate students from the University of Bologna (17 females) participated in this study. All participants were right-handed, native Italian speakers and reported normal or corrected-to-normal vision. All participants were naïve as to the purpose of the experiment.

### Apparatus and stimuli

The Experiment took place in a soundproof room. The participant sat in front of a 17” cathode-ray tube screen driven by a 1 GHz processor computer at a viewing distance of 50 cm. 

Participants were required to hold a mouse (Microsoft Wireless Notebook Laser Mouse 7000) with their right hand at a distance of 30 cm from the body (starting position). The subsequent towards or away movements were performed in a 60 cm long and 10 cm wide course on the table. This allowed participants to make a movement suitable for kinematics recording, namely allowing a displacement of the mouse of 30 cm in each direction (towards-away).

The E-Prime2 software controlled stimulus selection, response timing, and data collection. A black fixation cross (1.87° x 1.87° of visual angle) was presented at the beginning of each trial. The stimuli consisted of sentences written in black ink and presented at the centre of a white screen. Words were written in a 30-point size Courier New font. 

Half of the stimuli were composed by sensible sentences and the other half by non-sensible sentences (fillers). Both types of sentences were composed of two parts. The descriptive part referred to an object positively or negatively connoted by two different sets of proprieties, one related to its emotional object valence and the other to its graspability. Therefore, 16 different adjectives were used: 4 qualitative positive (e.g., attractive), 4 qualitative negative (e.g., ugly), 4 grasp-related positive (e.g., smooth) and 4 grasp-related negative (e.g., prickly). The action part was composed of an imperative verb implying a motion towards the self or towards another person and a pronoun referring to the object. An example of the sentence was “The object is attractive/prickly. Bring it to you/Give it to another person”. The order of the descriptive and action part was counterbalanced within subjects. 

With regard to the filler sentences, they had the same structure of the sensible sentences, with the exception of a non-sensible part. This non-sensible part could be either due to the adjective, i.e., “The object is tanned (/touchy), bring it towards you”, the verb, i.e., “The object is ugly, walk it to another person”, or the agent, i.e., “The object is smooth, give it to an eyelet”. For a complete list of sensible and filler sentences and their translation see the stimuli of Lugli et al’s [20] Experiment 1 at this link: http://laral.istc.cnr.it/borghi/Appendix_self_others_objects.pdf

The task consisted of determining whether each sentence was sensible or non-sensible. Participants were asked to position their right hand on the mouse and to move it towards or away from their body following a vertically traced course drawn on the tabletop. A vertical movement of the cursor always followed the movement of the mouse from the centrally presented sentence and by a congruent motion of the sentences, either towards or away from the participant's body [28,29]. The motion of the sentence was simulated by gradually increasing the font size and moving it slightly downwards (towards the participant’s body) or decreasing the font size and moving it upwards (away from the participant’s body). The mouse movement was coordinated with sentence displacement (i.e., velocity parameters were modified to slow down mouse velocity), so that the 30 cm. away or towards movements corresponded to reaching the upper or the lower part of the screen (i.e., the end of movement). The instructions stressed both the speed and the accuracy of performance. Participants were required to start the response movement as soon as the sentence sensibility judgment was made. Once participants had started the mouse movement, they were instructed to perform the movement with a natural velocity.

### Procedure

**Figure 1:**
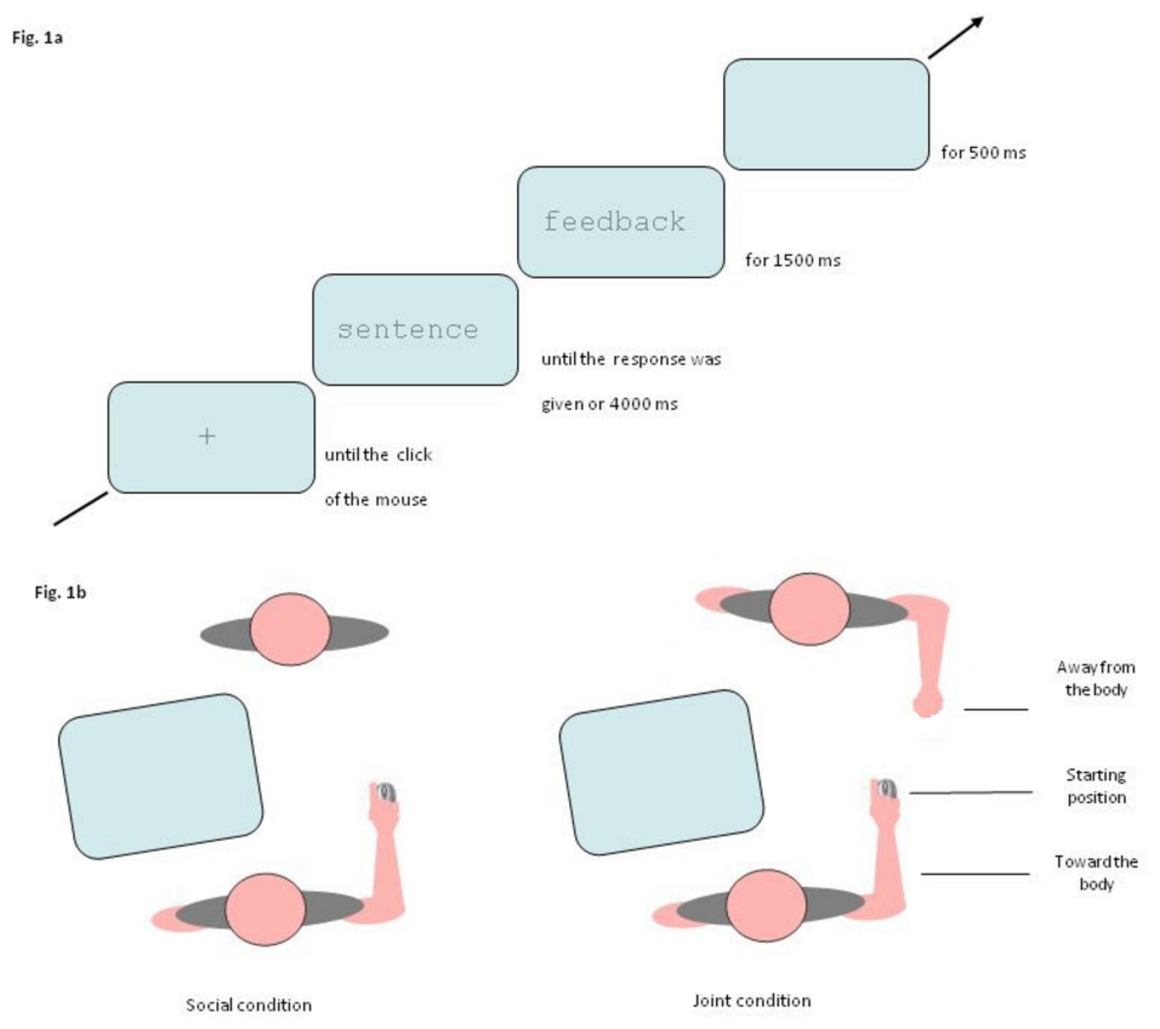
**a**. Sequence of events in a trial. At the start of each trial a fixation cross appeared in the center of the screen until participants clicked on it with the cursor of the mouse. The fixation cross was replaced by the sensible or non-sensible sentences until the response was given or until 4000 ms had expired. At response execution a 1500 ms feedback appeared. After a delay of 500 ms, the next trial was initiated. Note that stimuli are not drawn to scale. **b**. Example of the experimental setting for the Social and Joint conditions. In the Social condition (leftmost panel) the experiment sat in front of the participant and did not interact with him/her. In the Joint condition (rightmost panel) the experimenter interacted with the participant at the end task execution in order to reposition the mouse upon the starting position.

**Figure 2:**
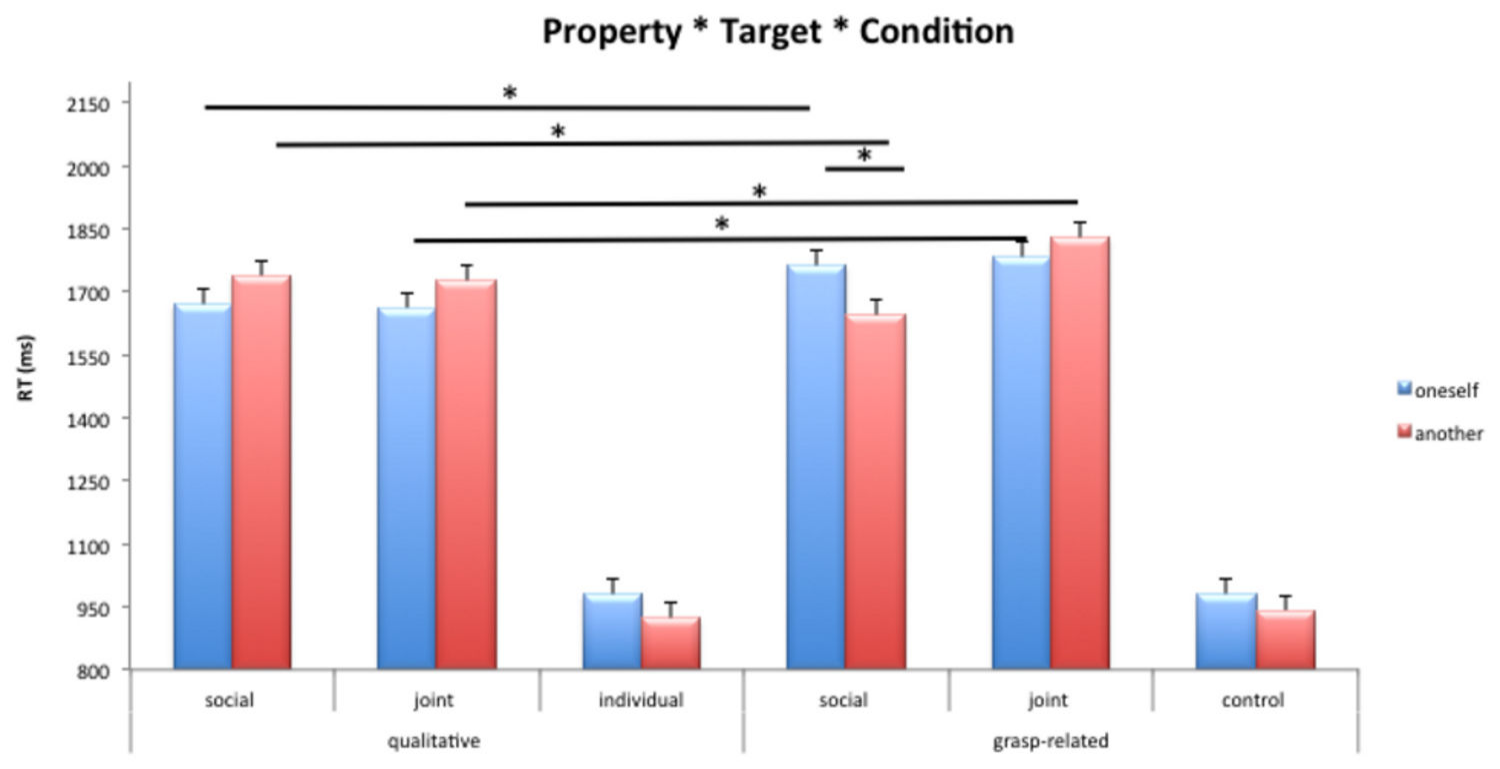
Mean RTs for qualitative and grasp-related properties. Bars are Standard Errors.

Participants were randomly assigned to one of the three conditions (Individual vs. Social vs. Joint). In all conditions, participants started each trial by clicking on the fixation cross. The sentence appeared, replacing the cross, and remained on the screen until participants provided a response or until 4000 ms had passed. In case of incorrect or delayed responses, the word “ERROR” or “DELAY”, respectively, appeared at the screen center in red uppercase letters for 1500 ms. After a blank screen of 500 ms in duration, the fixation cross appeared and a next trial was initiated (see Figure 1a). In the Individual and Social conditions participants executed this task without any interaction with the experimenter (see Figure 2). In the former condition the participant was indeed alone in the lab, while in the latter the experimenter sat in front of the participant as a mere observer without having any interaction (either verbal or motor) with him/her. In the Joint condition, the experimenter actively took part into the experiment: when responding with an away from the body movement, participants were required to leave the mouse away from their body, without returning in the central position. The task of the experimenter was to re-position the mouse back upon the central starting position. In this way the experimenter interacted with the participant only at the end of the task, that is, after the accomplishment of both the sensibility judgment and the response movement. In the Social and Joint condition the experimenter had a marker on her wrist, in a symmetrical position with respect to that of the participant, but her movements were not analyzed. 

Each condition was composed by two blocks of 64 trials. Each block was preceded by a short training phase (8 trials). The two blocks had opposite instructions: participants were required to move the mouse towards the body for sensible sentences and away from the body for fillers (block 1) or vice versa (block 2). The order of the blocks was balanced between subjects. 

### Data recording and kinematic analysis

Movements of the participants’ right hand were recorded using the 3D-optoelectronic SMART system (BTS Bioengineering, Milan, Italy). This system consists of four video cameras detecting infrared reflecting markers (spheres of 5-mm diameter) at a sampling rate of 120 Hz. Spatial resolution of the system is 0.3 mm. Recorded data were filtered using a linear smoothing low pass filter, i.e., a triangular filter where each value was the weighted mean computed over 5 samples (window duration 33.3 ms).

We used one marker applied on the wrist of the participant’s right hand. Participants were informed that their movements were recorded. We analyzed the time course of the participant’s wrist marker in order to study the spatio-temporal evolution of the response movement towards or away from the body. We decided to focus on a single parameter, that is the velocity peak, since it is known to be modulated by either social cues [26,30,31] and linguistic stimuli, for example action verbs [32]. 

The velocity peak corresponds to the maximum speed recorded on the wrist marker between the beginning of movement and its end. The movement we used is characterized by a single velocity peak, occurring during the accelerative phase of movement, (i.e. 50/60 % of movement time). 

RTs were also recorded together with the kinematics acquisition, in order to better define the temporal evolution of mouse movements. RTs were defined as the time between the click on the fixation cross and the beginning of the mouse movement. The start of the movement corresponded to the moment in which the mouse cursor moved 20 pixels from its starting point in a vertical direction. This measure combined a high sensitivity to true responses with a low responsiveness to small random mouse movements.

### Data analysis

The incorrect responses were removed from the analysis (2.7%). We also discarded all the filler sentences. Analyses of errors revealed no evidence of speed-accuracy trade-off, so we focused on RTs and kinematics analyses. 

Mean correct RTs and velocity peaks were submitted to a repeated-measures ANOVA with *Object Property* (grasp-related vs. qualitative), *Target* (oneself vs. another person), *Object Valence* (positive vs. negative) and *Movement Direction* (towards the body vs. away from the body) as the within-subject factors and *Condition* (Individual vs. Social vs. Joint) as the between-subjects factor. Fisher’s LSD post-hoc tests were conducted on significant interactions. 

We decided to present and discuss only the significant main effect and interactions involving the *Condition* factor, since it is the crucial variable for our hypotheses. 

## Results

### Reaction times (RTs)

The main effect of *Condition* was significant, *F*(2,21) = 27.91, MSe = 27800000, *p*<.001, *η*_*p*_^2^=.73. Post-hoc tests showed that the Individual condition was the fastest condition (*p*_s_<.01), and that the Social condition had a trend to be faster than the Joint one, *p* =.07 (all means are summarized in Table 1).

**Table 1:** Summary of mean RTs (ms) for the significant main effect of the *Condition* factor and its significant interactions.

CONDITION						
	social	joint	individual			
	1704	1749	956			
OBJECT VALENCE X CONDITION						
	social	joint	individual			
positive	1627	1721	973			
negative	1780	1778	939			
TARGET X CONDITION						
	social	joint	individual			
self	1716	1723	980			
other	1691	1776	932			
OBJECT PROPERTY X MOVEMENT X CONDITION						
	qualitative			grasp-related		
	social	joint	individual	social	joint	individual
near	1766	1676	956	1695	1753	994
far	1643	1711	946	1711	1858	929
OBJECT PROPERTY X TARGET X CONDITION						
	qualitative			grasp-related		
	social	joint	individual	social	joint	individual
self	1670	1662	980	1763	1783	981
other	1739	1725	922	1643	1828	942

The *Target* x *Condition* interaction was significant *F*(2,21) =5.01, MSe = 89600, *p*<.05, *η*_*p*_^2^=.32. Post-hoc tests indicated that either for the “oneself” and for the “another person” target RTs were faster in the Individual condition compared to the Social and Joint ones, *p*_*s*_<.001. Moreover, in the Individual condition participants responded faster when faced with sentences describing “another person” target (M = 932 ms) compared to the “oneself” one (M = 980 ms), *p*<.05. The opposite was true for the Joint condition since responses were faster when the target described was the “oneself” (M = 1723 ms) with respect the “another person” one (M = 1776), *p*<.05. The *Object Valence* x *Condition* interaction was significant*, F*(2,21) =7.88, MSe = 292000, *p*<.01, *η*_*p*_^2^=.43. Post-hoc tests showed that in the Individual condition faster RTs were yielded for both the positive and negative object valence with respect to the Social and Joint conditions (*p*_*s*_<.001). Only in the Social condition a significant difference between the positive and the negative object valence emerged (Ms= 1627 and 1780 ms, respectively, *p*<.05).

The *Object Property* x *Target* x *Condition* interaction was significant, *F*(2,21) =4.37, MSe = 94500, *p*<.05, *η*_*p*_^2^=.29, see Figure 2. Post-hoc tests showed that the Individual condition was the fastest (*p*_*s*_<.01) and that in the Social condition the grasp-related-“another person” combination yielded faster responses with respect to the grasp-related-“oneself” combination (*p*<.05). This same pattern did not emerge for the Joint condition (*p*=.26). In the Social condition, post-hoc tests indicated that: a) the qualitative-“oneself” combination was faster than the grasp-related-“oneself” one (*p*<.05), b) the grasp-related-“another person” combination yielded faster responses than the qualitative-“another person” combination (*p*<.05) and that c) the grasp-related-“another person” combination was faster than the grasp-related-“oneself” combination (*p*<.05 ). Finally, in the Joint condition, RTs were faster for the qualitative-“oneself” combination than for the grasp-related-“oneself” one (*p*<.05), and the responses to the qualitative-“another person” combination were faster than the ones for the grasp-related-“another person” combination (*p*<.05). 

The *Object Property* x *Movement direction* x *Condition* was significant, *F*(2,21) =3.72, MSe = 82700, *p*<.05, *η*_*p*_^2^=.26. The Individual resulted to be the fastest condition (*p*_*s*_<.01). In the Social condition, when sentences referred to qualitative proprieties, RTs were faster for the away-from-the-body movements than for the towards-the-body ones (*p*<.05). In the Joint condition, when participants were required to perform away-from-the-body movements, RTs were faster in response to qualitative proprieties compared to grasp-related ones (*p*<.05).

### Velocity Peak

**Figure 3:**
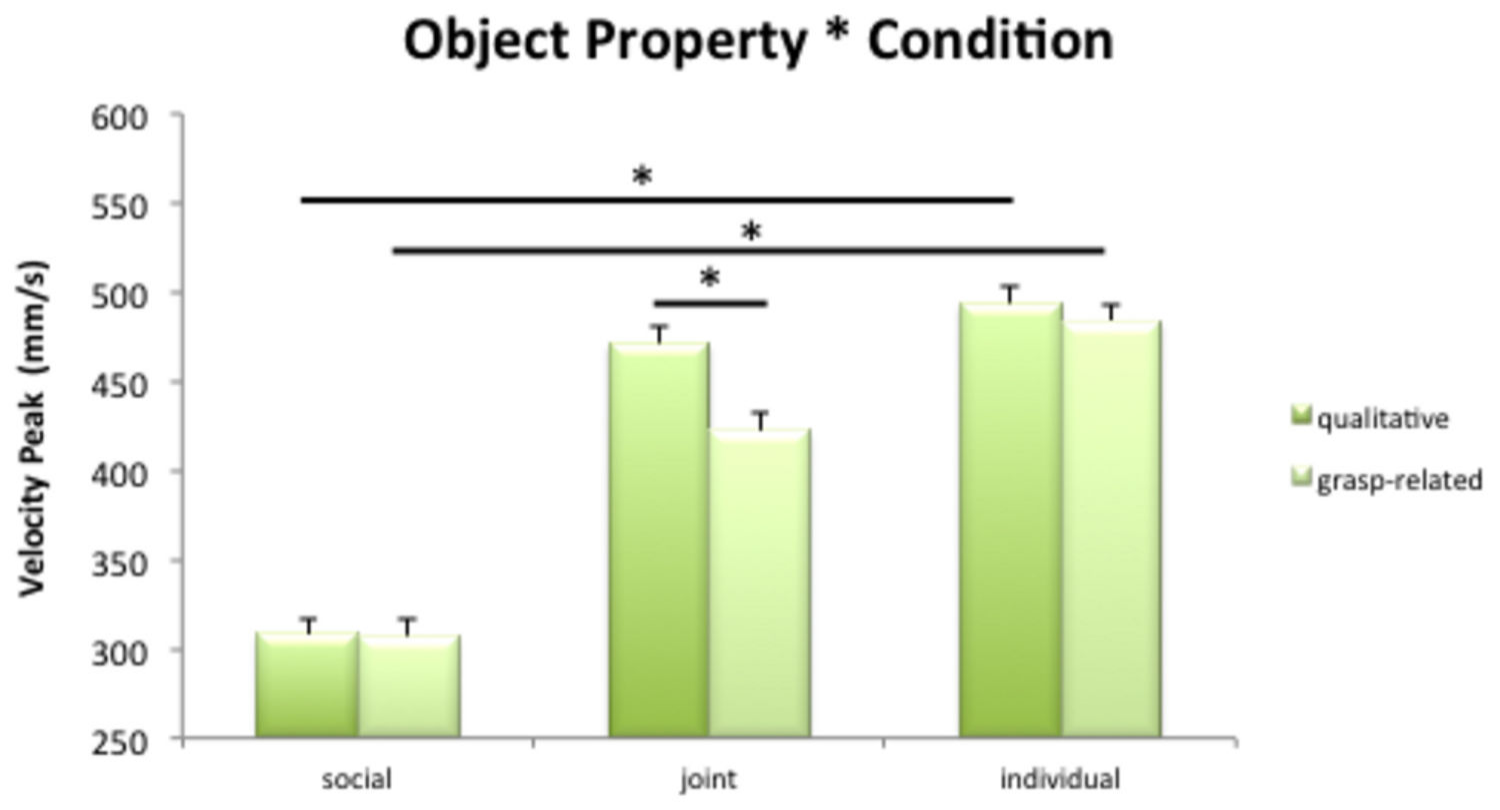
Mean velocity peaks for qualitative and grasp-related properties. Bars are Standard Errors.

Results on Velocity peaks showed that the *Object Property* x *Condition* interaction was significant, *F*(2,21) = 8.3, MSe = 18700, *p*<.01, *η*_*p*_^2^=.44, see Figure 3. Post-hoc tests indicated that the two object properties were differently perceived across conditions (all means are listed in Table 2). Only in the Joint condition, indeed, the velocity peaks for the two properties differed significantly, being higher for the qualitative than for the grasp-related ones (*p*<.01). Conversely, in the Social and Individual conditions the two properties did not differ (*p*_s_ > .05). Interestingly, differences between the Social and the Individual condition emerged when considering the two object properties separately. Velocity peaks for qualitative and for grasp-related properties were in fact higher in the Individual than in the Social condition (*p*_*s*_<.05). 

**Table 2:** Summary of mean velocity peaks (mm/s) for the significant main of the *Condition* factor and its significant interactions.

OBJECT PROPERTY X CONDITION			
	social	joint	individual
qualitative	308	471	494
grasp-related	307	422	484

## Discussion

The aim of this study was to investigate how a social experimental context would enhance the link between the sentence stimuli and the motor system, allowing participants to form a more detailed simulation of the linguistically described “another person” target. 

For this reason, we implemented three experimental conditions, in which the participants could perform the task alone (Individual condition), or in presence of the experimenter who acted as a mere observer (Social condition) or as a confederate (Joint condition). The direct comparison of these conditions gave us some additional insights in order to understand how implementing a social context could affect action sentence processing and thus overt movement execution, as showed by RTs and velocity peaks. Our main conclusions are listed below:

1. Observer vs. confederate

We confirmed our hypothesis that the presence of the experimenter during task execution affected the simulation of the targets and of the actions described by the linguistic stimuli. Insights on this point are given by the results on RTs, where the *Condition* factor resulted as significant, showing a slower performance when the experimenter acted as an observer (Social condition) and as a confederate (Joint condition), with respect to when she was absent (Individual condition). The same pattern emerged in the Condition x Target interaction. More specifically, we found that in the Joint condition RTs were slower when the linguistically described target was “another person” rather than “oneself”. The opposite was true, though, for the Individual condition. As hypothesized, these results showed that participants formed a detailed simulation of the “another person” target when the experimenter acted as a confederate. This is in line with the Indexical Theory and extends it, showing that the presence of a confederate contributed to the indexing of the words "another person". The fact that the effect was present with a confederate but not with a simple observer suggests that when activating the word referents we tend to adopt an action-based criterion. Similarly, we can consider how the ABL model gives account of our results, and how they can broaden the model. The setting of our study allowed participants to check whether their predictions formed during language comprehension are verified. When these predictions are confirmed, planned actions have to be realized, and more fine-grained motor control mechanisms are necessary. Thus, when the sentence stimuli referred to the "another person" target and the experimenter acted as a confederate, then social interaction became more demanding also in terms of motor control. As previously discussed, kinematics studies showed that interacting with others leads to an increase in accuracy, as the lower velocity peaks and the longer deceleration times reveal. Our data confirmed an increase in accuracy that is demonstrated by kinematics parameters and also by longer reaction times. This increase in accuracy is present only in case of successful grounding. 

The grounding mechanism at work when the simulation formed during language comprehension matches with the actual context has interesting implications for theories of social cognition as well. Traditional approaches would predict that the mere presence of another person, either acting as an observer or as a confederate, implies a social facilitation effect. This would be explained with the presence of others being a source of arousal, leading to an enhanced performance in a variety of tasks [33]. Our results disconfirm traditional approaches and are in line, instead, with Ideomotor Theories (e.g., 6) according to which participants’ performance is influenced only by the presence of a co-actor, particularly when his/her actions are similar to those the agent is able to perform [34] but not by the mere presence of an observer. Interestingly, recent evidence suggests that this matching process (probably mediated by the mirror neuron system) is present not only during imitative actions, but also when another person performs complementary actions that are part of the agent’s motor repertoire [35,36]. This is exactly what happened in our Joint condition. This matching mechanism is indeed not only implied in action observation and understanding, but is also at work during social interactions to support shared actions and coordination with other people.

2. Object properties: qualitative vs. grasp-related

Our results showed that the *Condition* factor significantly interacted with the *Object Property, Target* and *Movement direction* factors. Overall, results on RTs suggested that differences between the object properties and the type of target on one hand, and between the object properties and the movement direction on the other hand, differently impacted performance according to the presence of the experimenter as compared to the Individual condition. In fact, we found a RTs modulation only in the Social and Joint conditions. More specifically, the Social condition yielded faster responses for grasp-related properties when related to the “another person” target, that resulted faster as compared to “oneself” target, and when the object was described with qualitative proprieties. On the contrary, in the Joint condition RTs were faster: a) for the qualitative-“oneself” target combination than for the grasp-related-“oneself” one and b) for the grasp-related-“another person” combination with respect to the qualitative-“another person” one. These results suggested once more that the presence of the experimenter influenced our actions as they are thought to be more accurate as means of social interactions. 

Velocity peaks also gave interesting insights on this point: the Joint was the only condition that showed a significant difference between the two types of properties, with qualitative properties yielding higher velocity peaks with respect to the grasp-related properties. This is consistent with the idea that qualitative properties assume more relevance when an actual confederate is present, thus yielding overall faster responses. On the contrary, the two types of properties did not differ in the Social and Individual conditions. Differences between the Social and Individual conditions emerged only when considering the object properties separately: velocity peaks were indeed higher for qualitative and grasp-related properties in the Individual condition with respect to the Social one. This may suggest that responses to both object properties were slower in presence of an observer and this could have impacted movement execution at a general level without involving the processing of different object properties.

Overall, results on object properties confirm the hypothesis according to which the presence of an actual target, and particularly of a confederate, enhances response accuracy. The rationale of this prediction, advanced on the basis of previous kinematics evidence, is the following: given that we have to tune ourselves with another actual target in giving or obtaining something to/from her, we might pay more attention to fine-grained distinctions between object properties. This is exactly what we found. Moreover, velocity peaks analyses demonstrated that this sensitivity increased from the Individual to the Social to the Joint condition. The fact that the sensitivity to fine-grained differences of object properties did not emerge in the Individual condition as well might seem at odds with Ideomotor Theories, according to which action alternatives at the other's disposal might become represented and activate events representations that are functionally equivalent to the events representations used in one's own control of these actions. However, we think this is only partially the case, for at least two reasons. First, to the best of our knowledge, Ideomotor Theories focus on action representation, and the representation of fine-grained object properties is only indirectly related to action. Second, Ideomotor Theories can account for our finding that the differences between object properties played a major role in the presence of a confederate than of a simple observer. In this respect the predictions of the Ideomotor Theories are difficult to disentangle from those we advanced on the basis of previous kinematics evidence showing that interacting with another person improves movement accuracy, as it happens in our Joint condition. 

Below we will discuss further implications of our study, underlying what in our opinion are the novelties of our work with respect to the current literature. We will start with the methodological implications and then focus on the theoretical ones. 

Our results have implications for the relationship between a linguistically described and an actually experienced social context. In our study the presence of an actual target, i.e. the experimenter, probably allowed participants to instantiate the linguistically described “another person” target and this instantiation consistently changed the dynamics of the motor behaviour. Recent studies on spoken language comprehension with eye tracking paradigms monitored participant’s attention focusing on their gaze shifts (e.g., [37–40]; for a recent review [41]). Their results indicated that people incrementally inspect objects and characters once they are mentioned; in addition, the results showed that, on the basis of linguistic cues, participants anticipated relevant objects and characters in visual context. For example, when they heard “Pick up the cube”, they began to search for containers sufficiently large to accommodate the cube [38]. Overall, these studies indicated that the experimental context contributes in circumscribing the referential domain within which expressions are interpreted. However, these studies typically focused on spoken language and preferentially manipulated the presence of objects in a setting, rather than manipulating the social context. As far as we know, the present study is the first that aimed to verify whether the mere presence and the interaction with an actual target in the experimental setting would differently contribute to restricting the referential domain of the word “another person” and in changing the ways in which different objects properties are represented. These results might have theoretical implications for embodied cognition theories, as they help to refine the notion of simulation formed during language comprehension (for recent discussion on this notion, see 13,19,42). We believe that the investigation of the different ways in which linguistically activated motor simulation is mapped with the world we experience can represent a promising and novel line of research. The direct comparison of individual settings with social manipulations constitutes a methodological extension of previous studies in which linguistic and social contexts were manipulated separately. This is supported by the fact that only in presence of social setting the same linguistic stimuli proved to have an effect on overt motor behaviour. 

Further studies are needed to understand the dynamics underlying language grounding and, particularly, the relationship between linguistically described situations and actual ones. 

Finally, at a speculative level, our results cast doubts on the view according to which we automatically tend to have a collaborative attitude with others. Along this line, previous brain imaging studies showed that the motor resonance phenomenon, i.e., the tendency to tune our behavior to others’ behavior as reflected by the activation of the mirror neuron system, may be influenced by ethnic and cultural in group familiarity (e.g., 43,44). This evidence questioned the idea that the mirror system is automatically activated in presence of others in an imitative fashion; rather, it showed that the mirror system is modulated by the similarity between us and the others, as well as by the context [45]. In our case the mirror neuron system might be activated to comprehend the other’s action, but no automatic collaborative attitude was developed; rather, understanding the other’s action might have helped to prepare actions aimed at delimiting his/her influence. When we read a sentence referring to an unspecified other, the urge to perform actions favouring ourselves is milder than when an unknown co-actor is present; the latter case has an immediate impact on the way we respond to common objects. We become more accurate and pay more attention to the grasp-related characteristics of objects, given that a motor interaction has to occur. 
